# Lifetime Weight Characteristics of Adult Inpatients With Severe Anorexia Nervosa: Maximal Lifetime BMI Predicts Treatment Outcome

**DOI:** 10.3389/fpsyt.2021.682952

**Published:** 2021-07-15

**Authors:** Lisa-Katrin Kaufmann, Hanspeter Moergeli, Gabriella Franca Milos

**Affiliations:** ^1^Department of Consultation-Liaison Psychiatry and Psychosomatic Medicine, University Hospital Zurich, Zurich, Switzerland; ^2^Division of Neuropsychology, Department of Psychology, University of Zurich, Zurich, Switzerland

**Keywords:** anorexia nervosa, weight characteristics, hospitalization, weight suppression, treatment outcome

## Abstract

**Background:** The body mass index is a key predictor of treatment outcome in patients with anorexia nervosa. In adolescents, higher premorbid BMI is a strong predictor of a favorable treatment outcome. It is unclear whether this relationship holds true for adults with anorexia nervosa. Here, we examine adult patients with AN and investigate the lowest and highest lifetime BMI and weight suppression as predisposing factors for treatment outcome.

**Methods:** We included 107 patients aged 17–56 with anorexia nervosa and tracked their BMI from admission to inpatient treatment, through discharge, to follow-up at 1–6 years. Illness history, including lowest and highest lifetime BMI were assessed prior to admission. We used multiple linear regression models with minimal or maximal lifetime BMI or weight suppression at admission as independent variables to predict BMI at admission, discharge and follow-up, while controlling for patients' age, sex, and duration of illness.

**Results:** Low minimal BMI had a negative influence on the weight at admission, which in turn resulted in a lower BMI at discharge. Higher maximal BMI had a substantial positive influence on BMI at discharge and follow-up. Weight suppression was highly correlated with maximal BMI and showed similar effects to maximal BMI.

**Conclusion:** Our findings strongly support a relationship between low minimal lifetime BMI and lower BMI at admission, and between higher maximal lifetime BMI or weight suppression and a positive treatment outcome, even years after discharge. Overall, maximal BMI emerged as the most important factor in predicting the weight course in adults with AN.

## Introduction

Treatment for anorexia nervosa (AN) aims to restore and maintain a healthy body weight and to reduce the core psychopathology of the illness (1, 2), but long-term prognoses are oftentimes poor (3). The body mass index (BMI) is not only a key diagnostic measure of AN, but also a central measure of treatment outcome.

Premorbid BMI is assumed to be an important biological risk factor for the etiology of AN in adolescents, with lower premorbid BMI predicting the onset of AN (4). Previous studies in children and adolescents have suggested that higher premorbid weight acts as a protective factor for the onset of AN. For example, a large longitudinal study that tracked the BMI of children from birth to 12.5 years of age reported that the average growth trajectory of children with a subsequent onset of AN was lower than the trajectory of children who later did not develop an eating disorder (5). Premorbid BMI has been shown to be an important predictor of BMI at admission [e.g., (6, 7)]. In adolescents, higher premorbid BMI has been shown to be predictive of a favorable treatment outcome at discharge, at 1-year follow-up (6), and at 6–12-year follow-up (8). It is currently unclear if this relationship holds true for adult patients (9). In particular, it is unclear what role the longer duration of illness or the later onset of AN play with respect to the association of pretreatment weight characteristics and treatment outcome. The longer illness history of adult patients results in a more variable weight trajectory compared to adolescents. Premorbid BMI may not capture the complexity of trajectory and illness history. To account for this, the lowest and highest lifetime BMI can be used as key characteristics of past illness course.

While premorbid BMI is a measure of absolute weight status, weight suppression (the difference between highest adult weight and current *or* lowest weight) (10) represents a measure of relative weight status. Greater current weight suppression has been found to predict future onset of AN (11) and has been associated with faster and greater weight normalization during inpatient treatment of AN [e.g., (12, 13)]. However, there are mixed findings regarding long-term treatment outcomes, with reports of higher weight suppression at the time of lowest BMI being associated with higher BMI at 6- to 18-year follow-up (14), and higher weight suppression at discharge predicting better weight maintenance at 1-year follow-up (15), but also reports showing no effect of weight suppression at discharge on weight maintenance at 1-year follow-up (16).

Here, we examine BMI trajectories in adult patients with AN and investigate the lowest and highest lifetime BMI, and the weight suppression at the time of lowest BMI as predisposing factors for treatment outcome. Specifically, we examine the influence of minimal lifetime BMI, maximal lifetime BMI, and maximal weight suppression on the BMI at admission to inpatient treatment, at discharge, and at 1–6-year follow-up. Patients' age, sex, and duration of illness are considered as additional predictors.

## Methods

### Participants and Procedure

From January 2014 to December 2020, a total of 239 inpatients received psychiatric treatment at our eating-disorder unit, 181 of whom met the DSM-IV-TR criteria for AN during at least one of their stays. One hundred seven (59.1%) of the patients with AN had complete data and had given written informed consent to the analysis of their routinely collected data. Thus, the final sample included in this study consisted of 98 female and nine male patients. Illness history was assessed before admission to inpatient treatment, including minimal and maximal lifetime body mass index (BMI, kg/m^2^) and age at illness onset. Self-reported weights were verified using medical records. Weight-gain during treatment was measured at admission and at discharge as part of the regular treatment protocol. For patients with multiple stays during the study period, the cumulative duration of treatment, the BMI at first admission, and the BMI at last discharge were used. The reported age for all patients is the age at first admission and illness duration represents the time between illness onset and age at first admission. A subsample of 63 patients (female = 61, male = 2) participated in a follow-up. For the follow-up measurement, patients who had been discharged for at least 1 year were contacted by e-mail and telephone and asked to complete an online survey. As part of the survey, patients were asked to report their current weight and whether they had sought further treatment after discharge.

### Inpatient Treatment

All study participants were treated at our specialized eating-disorder unit. The inpatient treatment consists of a multimodal therapy programme with a target BMI ≥18.5 kg/m^2^, comprising individual and group psychotherapy, somatic controls and treatment, and structured nutrition increase, with the main goal of normalizing and stabilizing eating behavior and weight. Other therapeutic elements include body-perception therapy, art therapy, nutritional counseling, physiotherapy, and for patients who are advanced in the programme, vocational or educational training and cooking groups. Prior to admission, the indication for hospitalization and illness history is assessed in an detailed medical history interview. Minimal motivation and cooperation for voluntary therapy should be given as the admission to the unit is elective. All patients receive three main meals and three snacks per day with a fixed energy content ranging from 1,600 to 3,000 kcal/day depending on the treatment phase. Patients are required to participate in all elements of the treatment and to gain an average of 700 g/week until they reach the target BMI. Patients who are unable to adhere to the programme for several weeks have to discontinue therapy. However, as the overarching goal is to rehabilitate the patients as much as possible in their everyday lives, patients may complete treatment in several segments, taking breaks and resuming inpatient treatment at a later time. Between discharge and follow-up, the vast majority of patients (89%) received outpatient treatment in form of individual psychotherapy.

### Data Analysis

For the calculation of maximal and minimal lifetime BMI (maximal and minimal BMI hereafter), patients' height at admission and the recalled minimal and maximal lifetime weight after reaching current height were used. Maximal weight suppression was calculated as the difference between maximal BMI and BMI at admission.

For demographic and clinical data, mean, standard deviation (SD), and range are reported. Percentages are rounded to integers. To compare demographic and weight characteristics between female and male patients, Fisher's exact tests were used for the categorical characteristics and Wilcoxon rank sum tests were used to compare continuous characteristics. Bivariate Pearson correlations were calculated to examine the associations among BMI measures (results can be found in the Supplementary Material). To assess the predictive relevance of minimal BMI, maximal BMI, and maximal weight suppression for BMI at admission, at discharge, and at follow-up we fitted linear regression models, estimated using ordinary least squares. First we estimated a base model with the following prognostic parameters as independent variables: age at admission, duration of illness, sex, and BMI at admission (for the prediction of BMI at discharge) and BMI at discharge (for the prediction of BMI at follow-up). Next, minimal or maximal BMI or maximal weight suppression were added as predictors to the basic model to determine the additional variance they explained. To ensure robust estimations of regression coefficients, minimal and maximal BMI or maximal weight suppression were not entered in the same model due to collinearity. Analyses were conducted using R version 4.0.3 (17). All *p*-values are two-sided and were considered statistically significant at the 5% level.

## Results

### Sample Characteristics

Demographic and weight characteristics are summarized in Table 1. The female and male patients reported similar minimal and maximal BMI, maximal weight suppression, and a similar proportion of anorexia subtypes, with roughly 1/3 binge-purge and 2/3 restrictive. Female patients showed a slightly higher prevalence of depression compared to male patients (Table 1). During the study period, 38 patients (36%) were hospitalized more than once (up to five times).

**Table 1:** Demographic and weight characteristics.

	**Sex**		
	**Male** ***n* = 9**	**Female** ***n* = 98**	
**Variable**	**Mean (SD) [Range]/*n* (%)**	**Mean (SD) [Range]/*n* (%)**	***p*-value^a^**
Age (years)	24.14 (5.58) [17.19, 34.52]	24.86 (8.44) [17.00, 55.77]	0.9
Age at illness onset (years)	18.44 (3.88) [14.00, 24.00]	17.17 (5.84) [10.00, 46.00]	0.2
Illness duration (years)	5.70 (5.33) [1.15, 17.52]	7.71 (7.32) [0.50, 41.77]	0.5
BMI at admission	15.96 (1.33) [13.40, 18.20]	14.55 (1.65) [10.60, 18.30]	**0.023**
BMI at discharge	17.80 (1.61) [15.80, 20.10]	17.32 (1.84) [11.90, 20.40]	0.6
Min. BMI	14.19 (1.50) [11.00, 16.00]	13.52 (1.67) [10.00, 17.50]	0.15
Max. BMI	21.61 (3.49) [17.00, 28.00]	20.76 (3.43) [15.60, 39.00]	0.4
Weight suppression	5.66 (3.03) [1.70, 11.90]	6.21 (3.67) [0.20, 22.40]	0.6
AN type			>0.9
binge-purge	3 (33%)	34 (35%)	
restrictive	6 (67%)	64 (65%)	
Comorbid depression	2 (22%)	58 (59%)	**0.041**

a *Wilcoxon rank sum test; Fisher's exact test. In bold, p-values < 0.05*.

### BMI at Admission

#### Relationship Between Minimal/Maximal BMI, Weight Suppression and BMI at Admission

We performed a multiple regression analysis in which the dependent variable was BMI at admission while the independent variables were age, sex, and duration of illness (base model). The model explained a weak proportion of variance (adj. R^2^ = 0.06). Adding minimal BMI to the base model significantly improved the prediction [F_(1, 102)_ = 60.95, *p* < 0.0001], explaining a substantial proportion of variance (adj. R^2^ = 0.41). Within this model the effect of minimal BMI was significantly positive (Table 2). Adding maximal BMI to the base model did not improve the prediction [F_(1, 102)_ = 0.34, *p* = 0.562]. Adding weight suppression to the base model significantly improved the prediction [F_(1, 102)_ = 20.87, *p* < 0.0001], explaining a moderate proportion of variance (adj. R^2^ = 0.21). Within this model the effect of weight suppression was significantly negative (Table 2).

**Table 2:** Summary of regression models for BMI at admission, discharge and follow-up.

**Variable**	**Base model**			**Min. BMI**			**Max. BMI**			**Weight suppression**		
	**Beta**	**95% CI^a^**	***p*-value**	**Beta**	**95% CI^a^**	***p*-value**	**Beta**	**95% CI^a^**	***p*-value**	**Beta**	**95% CI^a^**	***p*-value**
**Admission**												
Age (years)	0.06	0.00, 0.11	0.051	0.01	−0.04, 0.05	0.70	0.05	−0.01, 0.11	0.11	0.09	0.04, 0.14	**0.001**
Illness duration (years)	−0.05	−0.11, 0.02	0.14	0.01	−0.04, 0.07	0.60	−0.04	−0.11, 0.02	0.20	−0.06	−0.12, 0.00	**0.046**
Sex	−1.40	−2.5, −0.23	**0.019**	−1.00	−1.9, −0.13	**0.025**	−1.30	−2.5, −0.20	**0.021**	−1.20	−2.3, −0.22	**0.018**
Min. BMI				0.62	0.46, 0.78	** <0.001**						
Max. BMI							0.03	−0.07, 0.13	0.60			
Weight suppression										−0.19	−0.27, −0.11	** <0.001**
R^2^ (adj. R^2^)		0.09 (0.06)	**0.021**		0.43 (0.41)	** <0.001**		0.09 (0.06)	**0.04**		0.24 (0.22)	** <0.001**
**Discharge**												
Age (years)	−0.01	−0.07, 0.05	0.80	−0.02	−0.07, 0.04	0.60	−0.03	−0.09, 0.03	0.30	−0.03	−0.09, 0.03	0.30
Illness duration (years)	−0.02	−0.08, 0.05	0.50	−0.01	−0.08, 0.06	0.80	−0.01	−0.07, 0.06	0.80	−0.01	−0.07, 0.06	0.80
Sex	0.31	−0.84, 1.5	0.60	0.27	−0.88, 1.4	0.60	0.39	−0.74, 1.5	0.50	0.39	−0.74, 1.5	0.50
BMI at admission	0.53	0.34, 0.73	** <0.001**	0.44	0.20, 0.69	** <0.001**	0.52	0.33, 0.71	** <0.001**	0.64	0.43, 0.85	** <0.001**
Min. BMI				0.15	−0.10, 0.40	0.20						
Max. BMI							0.11	0.02, 0.21	**0.022**			
Weight suppression										0.11	0.02, 0.21	**0.022**
R^2^ (adj. R^2^)		0.24 (0.21)	** <0.001**		0.25 (0.21)	** <0.001**		0.28 (0.24)	** <0.001**		0.28 (0.24)	** <0.001**
**Follow-up**												
Age (years)	0.03	−0.09, 0.16	0.60	−0.01	−0.14, 0.13	>0.90	−0.07	−0.22, 0.08	0.40	−0.07	−0.22, 0.08	0.40
Illness duration (years)	−0.05	−0.19, 0.08	0.40	−0.01	−0.15, 0.14	>0.90	0.02	−0.13, 0.16	0.80	0.02	−0.13, 0.16	0.80
Sex	−1.90	−4.6, 0.82	0.20	−2.30	−5.0, 0.42	0.10	−2.00	−4.6, 0.64	0.14	−2.00	−4.6, 0.64	0.14
BMI at admission	0.15	−0.22, 0.53	0.40	−0.06	−0.51, 0.38	0.80	0.13	−0.23, 0.49	0.50	0.38	−0.04, 0.79	0.074
BMI at discharge	−0.03	−0.43, 0.37	0.90	−0.06	−0.45, 0.33	0.80	−0.09	−0.48, 0.30	0.60	−0.09	−0.48, 0.30	0.60
Min. BMI				0.42	−0.07, 0.90	0.089						
Max. BMI							0.25	0.03, 0.46	**0.028**			
Weight suppression										0.25	0.03, 0.46	**0.028**
R^2^ (adj. R^2^)		0.07 (0.01)	0.48		0.12 (0.03)	0.28		0.15 (0.06)	0.15		0.15 (0.06)	0.15

a *CI, Confidence Interval. In bold, p-values < 0.05*.

### BMI at Discharge

#### Treatment Outcome

**Figure 1:**
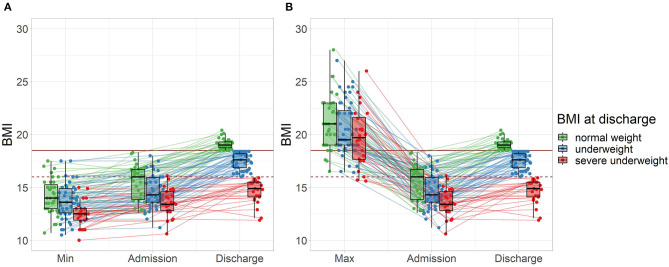
Treatment outcome and lifetime weight characteristics of all patients (*n* = 107) grouped by BMI at discharge. **(A)** Minimal lifetime BMI. **(B)** Maximal lifetime BMI (two patients with a maximal BMI >30 are not displayed). The horizontal mark of the boxplots signifies the median, edges of the box represent 25 and 75th percentiles, and the whiskers extend to 1.5 interquartile ranges. Normal weight: BMI ≥18.5 kg/m^2^, represented by the solid horizontal line, severe underweight: BMI <16.0 kg/m^2^, represented by the dashed horizontal line.

At discharge, 33% of patients had reached normal weight with a BMI ≥18.5 kg/m^2^ (good treatment outcome), while 67% percent were still underweight (intermediate treatment outcome), including 23% which were severely underweight (BMI <16.0 kg/m^2^, poor treatment outcome). The proportion of underweight patients was similar between female and male patients (all *p* > 0.80). Interestingly, the subgroup with severe underweight at discharge showed a history of severe underweight in minimal BMI (Figure 1A), whereas a less clear picture emerged for maximal BMI (Figure 1B).

#### Relationship Between Minimal/Maximal BMI, Weight Suppression and BMI at Discharge

We performed a multiple regression analysis in which the dependent variable was BMI at discharge while the independent variables were age, sex, duration of illness, and BMI at admission (base model). The model explained a moderate proportion of variance (adj. R^2^ = 0.21). Within this model the effect of BMI at admission was significantly positive (Table 2). Adding minimal BMI to the model did not improve the prediction [F_(1, 101)_ = 1.451, *p* = 0.231]. Adding maximal BMI to the base model significantly improved the prediction [F_(1, 101)_ = 5.412, *p* = 0.022], explaining a substantial proportion of variance (adj. R^2^ = 0.24). Within this model the effect of BMI at admission (beta = 0.52, 95% CI [0.33, 0.71], t_(101)_ = 5.39, *p* < 0.001) and the effect of maximal BMI were significantly positive (Table 2). Adding weight suppression at admission to the base model did improve the prediction [F_(1, 101)_ = 5.41, *p* = 0.022]. The model explained a significant and substantial proportion of variance (adj. R^2^ = 0.24). Within this model, effect of weight suppression at admission was significantly positive (Table 2).

### BMI at Follow-Up

#### Follow-Up Outcome

**Figure 2:**
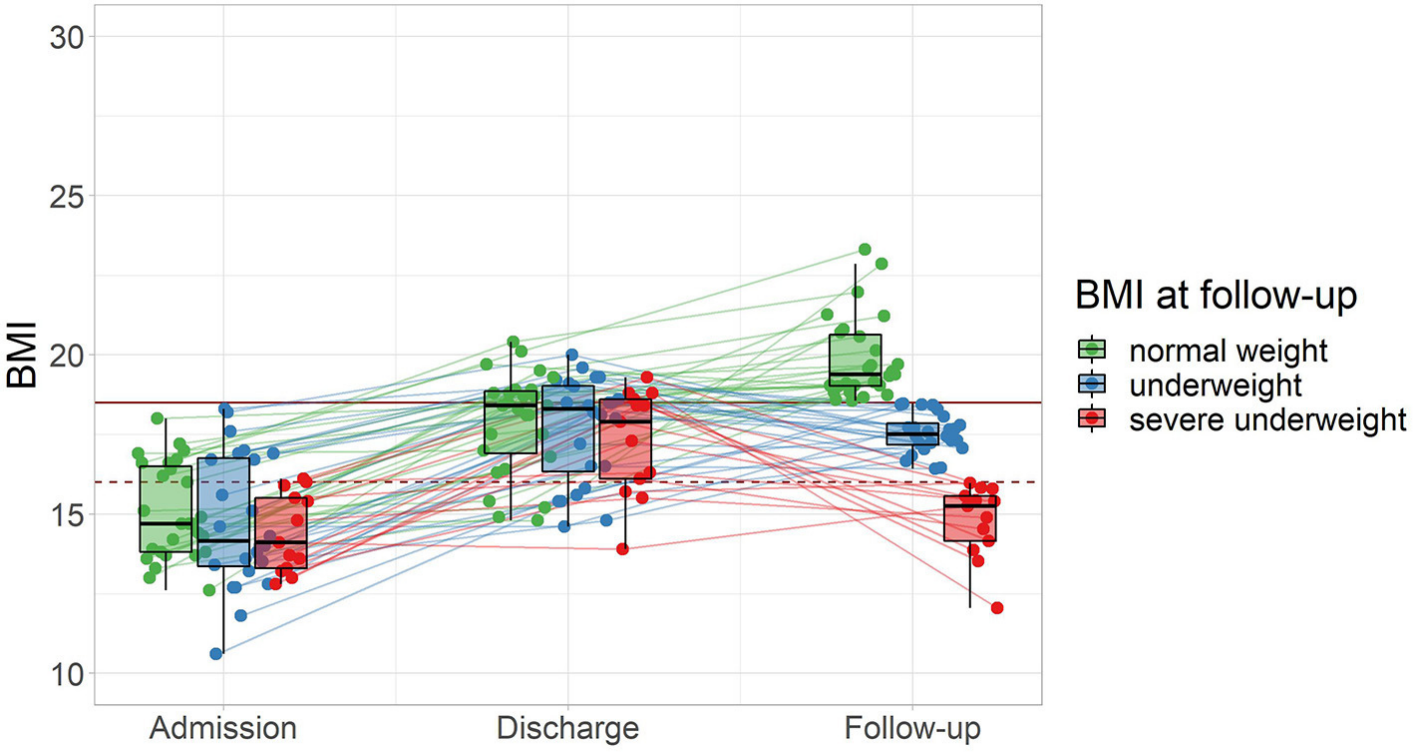
Follow-up outcome and weight trajectories of patients from admission to follow-up (*n* = 63) grouped by BMI at follow-up. The horizontal mark of the boxplots signifies the median, edges of the box represent 25 and 75th percentiles, and the whiskers extend to 1.5 interquartile ranges. Normal weight: BMI ≥18.5 kg/m^2^, represented by the solid horizontal line, severe underweight: BMI <16.0 kg/m^2^, represented by the dashed horizontal line.

Within the subsample of 63 patients who participated in the follow-up, the women reported a lower average BMI at follow-up (mean = 17.85 (2.12), [12.05, 22.86]) compared to the men (mean = 21.48 (2.57), [19.67, 23.30], *p* = 0.043). Follow-up took place after an average of 2.89 years (SD = 1.45, range = [1.00, 5.90]). At follow-up, 42% of patients reported a BMI ≥18.5 kg/m^2^. Of the subsample, 19% had maintained a BMI ≥18.5 kg/m^2^, 23% had reached a BMI ≥18.5 kg/m^2^ after discharge, while another 23% had lost weight and returned to underweight (BMI <18.5 kg/m^2^), and 34% were underweight at discharge as well as follow-up. Separating the patients into normal weight (BMI ≥18.5 kg/m^2^), underweight (BMI <18.5 kg/m^2^), and severe underweight BMI (<16.0 kg/m^2^) by their BMI at follow-up, there was no evidence for differences between these subgroups of BMI at admission or discharge (Figure 2).

#### Relationship Between Minimal/Maximal BMI, Weight Suppression and BMI at Follow-Up

Finally, we performed a multiple regression analysis in which the dependent variable was BMI at follow-up while the independent variables were age, sex, duration of illness, BMI at admission, and BMI at discharge (base model). The model explained a non-significant and weak proportion of variance (adj. R^2^ = −0.007). Adding minimal BMI to the model did not improve the prediction [F_(1, 57)_ = 3.00, *p* = 0.089]. Adding maximal BMI to the base model significantly improved the prediction [F_(1, 57)_ = 5.09, *p* = 0.028], however the model explained only a non-significant proportion of variance (adj. R^2^ = 0.06). Within this model the effect of maximal BMI was significantly positive (Table 2). Adding weight suppression at admission to the base model did improve the prediction [F_(1, 57)_ = 5.09, *p* = 0.028]. The model explained a not significant and moderate proportion of variance (adj. R^2^ = 0.06). Within this model, effect of weight suppression at admission was significantly positive (Table 2). Similar multiple regression results were seen for all follow-up models when including time between discharge and follow-up (time to follow-up) as covariate. Time to follow-up did not significantly alter the model predictions (all *F* < 1.44, *p* > 0.24) and had no significant effect on BMI at follow-up (all *t* < 1.20, *p* > 0.24).

## Discussion

The BMI is a critical marker of illness severity in AN and is widely considered a key predictor of treatment outcome in adolescent, however detailed analysis of the predictive value of BMI history in adult patients has been lacking. In the present study, we examined the lowest and highest lifetime BMI, and the weight suppression at admission as predisposing factors for the outcome of inpatient treatment in adult patients with AN. Specifically, we analyzed the relationship of minimal BMI, maximal BMI, and maximal weight suppression with the BMI at admission, discharge, and follow-up, while controlling for other parameters of illness history.

Our results showed a strong association of minimal lifetime BMI and BMI at admission, even when considering patients' age, sex, and duration of illness. An increment of 1.0 kg/m^2^ in minimal BMI was associated with a mean increase of 0.62 kg/m^2^ in BMI at admission. Higher weight suppression contributed moderately to the prediction of lower BMI at admission when controlling for age, sex, and duration of illness, whereas maximal BMI had no predictive power for the BMI at admission. This indicates, similar to the premorbid BMI in adolescents (6, 18), that minimal lifetime BMI is a strong predictor for the weight status at admission in adults. For the BMI at discharge, BMI at admission and the parameters of illness history together explained 21% of the variance, with BMI at admission being the strongest outcome predictor. Minimal BMI added little information to this. However, maximal BMI and weight suppression improved this prediction independently of BMI at admission, with a 1.0 kg/m^2^ increase in maximal BMI or weight suppression being associated with a 0.11 kg/m^2^ increase in BMI at discharge. The counterintuitive association of higher weight suppression as beneficial predictor is consistent with previous reports of a positive association of weight suppression and weight gain during inpatient (12, 13, 19) and outpatient treatment (20). Given the high correlation between weight suppression and maximal BMI, it stands to reason that the beneficial effect of weight suppression is driven by maximal BMI.

Finally, the BMI at follow-up was not predictable by BMI at admission or BMI at discharge. Minimal BMI was significantly correlated with BMI at follow-up, but added no additional information when controlling for the other variables. However, higher maximal BMI or weight suppression of 1.0 kg/m^2^ was associated with a 0.25 kg/m^2^ increase in BMI at follow-up. The lack of predictive power of the BMI at discharge is in contrast to reports of a 6-month follow-up (21), however this difference might be explained by the longer time to follow-up in our study. Consistent with our results, the above-mentioned study reports low predictive power for the minimal BMI (21). Maximal BMI itself has not been considered as predictor of follow-up BMI in previous research, but appears to be the driving force behind weight suppression at admission given their high correlation. The positive predictive power of weight suppression is in line with reports on adolescents with AN (14), where greater weight suppression at lowest BMI predicted higher BMI at 6-, 10-, and 18-year follow-up.

Taken together, a low minimal lifetime BMI seems to have a negative influence on the weight at admission, which in turn results in a lower BMI at discharge. Higher maximal BMI had a positive influence on BMI at discharge, and at follow-up maximal BMI had become more important than BMI at admission or discharge, contributing significantly to a higher weight. Overall, maximal BMI emerged as the most important factor in predicting the course of AN. While the underlying mechanism for this is unclear, lower maximal BMI may reflect metabolic aspects of the illness, such as a genetic predisposition to lower body fat, which is known to contribute to the etiology of AN (22, 23). From a clinical point of view, our therapeutic experience suggests that a maximal lifetime BMI within a normal range can positively influence the course of weight gain treatment. It is conceivable that for patients who have had body experiences with weight in the normal range, therapeutic weight gain up to a know weight is more imaginable and thus easier to achieve.

In recent years, the concept of the weight suppression, as the difference between maximal BMI and current or lowest BMI, has gained attention. Our results support the notion that greater weight suppression at admission is associated with higher BMI at discharge and better weight maintenance at follow-up. Considering the constituents of weight suppression that may drive its predictive power (10), it is apparent that maximal lifetime BMI is the key factor in the present study. Therefore, given the law of parsimony (Occam's razor), it seems most important to determine the maximal BMI in order to predict treatment outcome and BMI at follow-up in patients with AN.

The longer duration of illness in our adult sample did not emerge as meaningful predictors of treatment outcome or outcome at follow-up. While duration of illness is a known influence on long-term trajectories of AN [e.g., (3, 24)], this is in line with follow-up reports assessing treatment outcome at 6-month (21) and 1-year follow-up (6). Of concern, although in line with the literature (25–27), is that more that the half of the patients had remained underweight or returned to underweight at follow-up. These troubling findings underline the need of new therapeutic strategies to better treat severely ill patients and prevent relapse, and intensify research in this field (28).

One of the limitations of the present study is that a direct comparison between minimal BMI, maximal BMI and premorbid BMI is only partially possible. However, in adult samples, it is often not possible to determine the premorbid BMI since information on height at illness onset are not available. Instead, the minimal and maximal lifetime BMI are readily available data that are well-remembered by patients. Minimal and maximal lifetime BMI were assessed using self-reported values from the medical history interview. While all information was carefully checked against the medical records, biased reporting cannot fully be ruled out. However, as weight is inherently a key information to anorexia nervosa, patients are very accurate in reporting their weights (29, 30). The present study focussed on BMI as measure of treatment and follow-up outcome. While BMI is a core outcome measure in AN, the authors note that psychiatric and psychological aspects also play an important role and should be considered in future studies.

To conclude, our results suggest that a lower minimal lifetime BMI presents a negative prognostic factor in the short-term, promoting a lower BMI at admission. In contrast, a higher maximal lifetime BMI proved to be a positive prognostic factor in the medium and long-term, promoting better treatment outcomes even years after discharge. In addition, the very high correlation between maximal BMI and weight suppression at admission emphasizes the role of maximal BMI in weight trajectories. These findings highlight the importance of considering both the lower and especially the upper end of the lifetime weight range when treating adult patients with severe AN.

## Data Availability Statement

The raw data supporting the conclusions of this article will be made available by the authors, without undue reservation.

## Ethics Statement

The studies involving human participants were reviewed and approved by Kantonale Ethikkommission Zürich. The patients/participants provided their written informed consent to participate in this study.

## Author Contributions

L-KK: conceptualization, data curation, formal analysis, visualization, writing–original draft, and review & editing. HM: formal analysis and writing–review & editing. GM: conceptualization, writing–review & editing, and funding acquisition. All authors contributed to the article and approved the submitted version.

## Conflict of Interest

The authors declare that the research was conducted in the absence of any commercial or financial relationships that could be construed as a potential conflict of interest.
