# Supplementary material for: Lifetime Weight Characteristics of Adult Inpatients With Severe Anorexia Nervosa: Maximal Lifetime BMI Predicts Treatment Outcome
Source: Front Psychiatry. 2021 Jul 15;12:682952. doi: 10.3389/fpsyt.2021.682952 (PMC8319499; doi:10.3389/fpsyt.2021.682952)
Supplement: Supplementary file 1 [file Table_1.docx]

**Supplement: Lifetime weight characteristics of adult inpatients with anorexia nervosa**

# SUPPLEMENTAL RESULTS

### Relationship between minimal/maximal BMI, weight suppression and BMI at admission

We examined the relationships of minimal and maximal BMI, and maximal weight suppression with the BMI at admission using bivariate correlation analyses (Table S1). Minimal BMI was significantly correlated with BMI at admission, explaining 40% of variance, and negatively correlated with weight suppression (r = -0.34). Maximal BMI showed no significant correlation with BMI at admission (Table S1).

### Relationship between minimal/maximal BMI, weight suppression and BMI at discharge

The bivariate correlation analyses showed significant correlations between BMI at discharge and BMI at admission (23% variance explained), minimal BMI (15% variance explained), and maximal BMI (4% variance explained), while weight suppression was not significantly correlated with BMI at discharge (Table S1).

**Table S1.** *Correlation analyses between measures of BMI (n = 107)*

| Variable | BMI at admission | BMI at discharge | Min. BMI | Max. BMI | Weight suppression |
| --- | --- | --- | --- | --- | --- |
| **BMI at admission** | - |  |  |  |  |
| **BMI at discharge** | 0.48**** | - |  |  |  |
| **Min. BMI** | 0.63**** | 0.39**** | - |  |  |
| **Max. BMI** | 0.13 | 0.21* | 0.30** | - |  |
| **Weight suppression** | -0.34*** | -0.02 | -0.01 | 0.89**** | - |
| Note. *p<.05, **p<.01, ***p<.001, ****p<.0001 | | | | | |

### Relationship between minimal/maximal BMI, weight suppression and BMI at follow-up

The bivariate correlation analyses showed significant correlations between BMI at follow-up and

minimal BMI, and maximal BMI, and BMI at discharge (Table S2). BMI at admission, BMI at discharge, and weight suppression were not significantly correlated with BMI at follow-up (Table S2).

**Table S2.** *Correlation analyses between measures of BMI (n = 63)*

| Variable | BMI at admission | BMI at discharge | BMI at follow-up | Min. BMI | Max. BMI | Weight suppression | Time to follow-up |
| --- | --- | --- | --- | --- | --- | --- | --- |
| **BMI at admission** | - |  |  |  |  |  |  |
| **BMI at discharge** | 0.47**** | - |  |  |  |  |  |
| **BMI at follow-up** | 0.16 | 0.1 | - |  |  |  |  |
| **Min. BMI** | 0.61**** | 0.36** | 0.27* | - |  |  |  |
| **Max. BMI** | 0.17 | 0.18 | 0.25* | 0.43*** | - |  |  |
| **Weight suppression** | -0.34** | -0.06 | 0.16 | 0.1 | 0.87**** | - |  |
| **Time to follow-up** | -0.08 | -0.05 | 0.01 | -0.23 | 0.11 | 0.14 | - |
| Note. *p<.05, **p<.01, ***p<.001, ****p<.0001 | | | | | | | |
